# Setting for “Normal” Serum Ferritin Levels in Patients with Transfusion-Dependent Thalassemia: Our Current Strategy

**DOI:** 10.3390/jcm10245985

**Published:** 2021-12-20

**Authors:** Anna Spasiano, Antonella Meloni, Silvia Costantini, Emilio Quaia, Filippo Cademartiri, Patrizia Cinque, Alessia Pepe, Paolo Ricchi

**Affiliations:** 1 Unità Operativa Semplice Dipartimentale Malattie Rare del Globulo Rosso, Azienda Ospedaliera di Rilievo Nazionale “A. Cardarelli”, 80131 Napoli, Italy; spasiano.anna@tiscali.it (A.S.); silvia.costantini@aocardarelli.it (S.C.); patrizia.cinque@aocardarelli.it (P.C.); 2 Cardiovascular and Neuroradiological Multimodality Unit, Fondazione G. Monasterio CNR-Regione Toscana, 56124 Pisa, Italy; antonella.meloni@ftgm.it (A.M.); fcademartiri@ftgm.it (F.C.); 3 U.O.C. Bioingegneria e Ingegneria Clinica, Fondazione G. Monasterio CNR-Regione Toscana, 56124 Pisa, Italy; 4 Institute of Radiology, Department of Medicine, University of Padua, 35122 Padua, Italy; emilio.quaia@unipd.it (E.Q.); alessia.pepe@ftgm.it (A.P.)

**Keywords:** serum ferritin, thalassemia, iron overload, magnetic resonance imaging

## Abstract

This cross-sectional study aimed to establish the association between serum ferritin levels and organ iron overload (IO) and overall morbidity in transfusion-dependent thalassemia (TDT) patients. One hundred and three TDT patients (40.03 ± 9.15 years; 57.3% females) with serum ferritin < 2500 ng/mL were included. IO was assessed by T2* magnetic resonance imaging. Three groups were identified based on mean serum ferritin levels: <500 ng/mL (group 0; N = 32), 500–1000 ng/mL (group 1; N = 43), and 1000–2500 ng/mL (group 2; N = 28). All demographic and biochemical parameters were comparable among the three groups, with the exception of the triglycerides being significantly lower in group 0 than in group 2. No difference was found in the frequency of hepatic, endocrine, and cardiac complications. Hepatic IO was significantly less frequent in group 0 versus both groups 1 and 2. No patient with a serum ferritin level < 500 ng/mL had significant myocardial IO and alterations in the main hematological parameters. No difference in the distribution of the different chelation regimens was found. Serum ferritin < 500 ng/mL appears to be achievable and safe for several TDT patients. This target is associated with the absence of significant cardiac iron and significantly lower hepatic IO and triglycerides that are well-demonstrated markers for cardiac and liver complications.

## 1. Introduction

Iron chelation therapy is still a vital and fundamental therapy for reducing and preventing cardiac, hepatic, and endocrine complications induced by iron overload (IO) in patients with various chronic transfusion-dependent anemias [1,2]. The optimal management of patients with transfusion-dependent thalassemia (TDT) requires vigilant monitoring of body iron, particularly in the liver and in the heart. Although extra-cardiac and extra-hepatic IO are less crucial to overall survival, they could play a clinically relevant role to other morbidities [3,4]. Therefore, several studies involving TDT patients have focused mainly on the effects of chelation therapies on hepatic and myocardial iron overload [5,6,7,8,9,10], but an increasing body of evidence is prompting attention to the quantification of pancreatic iron overload as a potential source of endocrinopathies [2,11,12].

On the other hand, serum ferritin measurement is still a repeatable and low-cost method to roughly assess both iron overload and the effectiveness of iron chelation therapy. However, in the past, a great body of research was directed to establishing a relationship between serum ferritin and body iron stores, particularly in the presence of a considerable iron burden. Serum ferritin levels correlate significantly with liver iron concentration (LIC), and in the management of patients with TDT, an imperative issue is to maintain serum ferritin values < 1000 ng/mL because they are associated with higher survival rates and improved heart and liver function [3,13,14,15]. On the other hand, because of the risk of over-chelation, there are still concerns about reaching serum ferritin levels < 1000 ng/mL, the point that the dose reduction in desferrioxamine (DFO) is recommended by most guidelines [16]. Similarly, in deferasirox (DFX) therapy, dose reduction is still suggested for ferritin values < 1000 ng/mL.

Nowadays in TDT patients, the full understanding of the need for optimal chelation therapy and tailored application of different available chelation regimens is leading to the approach of lower and lower ferritin levels and iron burden. Therefore, in specialized centers for hemoglobinopathies, there is an increasing opportunity to reach serum ferritin below 1000 ng/mL and normal body iron stores across different chelation regimens and to evaluate their safety and the risk of over-chelation.

The aim of this study was to evaluate cross-sectionally the association between ferritin levels, organs’ IO, and overall morbidity in a large cohort of TDT patients followed in a single center specialized in the care of hemoglobinopathies.

## 2. Materials and Methods

### 2.1. Study Population

Among the 114 TDT patients followed at the Rare Blood Cell Disease Unit, AORN Cardarelli Hospital in Naples, Italy, we selected those with a mean serum ferritin value < 2500 ng/mL in the last year.

All patients were regularly transfused to maintain a pretransfusion hemoglobin concentration above 9–10 g/dl and were regularly chelated.

Eighty-five patients underwent an MRI scan within three months from the last blood test within the Extension-Myocardial Iron Overload in Thalassemia (E-MIOT) Network, where MRI exams are performed using homogeneous, standardized, and validated procedures for the heart, liver, and pancreas [17,18,19].

The study complied with the Declaration of Helsinki. All patients gave written, informed consent to the protocol. The study was approved by the Ethics Committee.

### 2.2. Biochemical Assays

Blood samples were taken in the morning, at least two weeks after the blood transfusions. All biochemical parameters were assessed in the same laboratory using commercially available kits.

Serum ferritin levels were assayed using a Microparticle Enzyme Immunoassay (MEIA) Imx Ferritin kit (Abbott, Chicago, IL, USA). The average of a 12-month observation period around the MRI scan assessment was considered.

### 2.3. Iron Overload Assessment

MRI scans were performed on conventional clinical 1.5T scanners using phased-array receiver surface coils.

A mid-transverse hepatic slice [20], five or more axial slices including the whole pancreas [21], and three parallel short-axis views (basal, medium, and apical) of the left ventricle (LV) [17,22] were acquired with T2* multiecho gradient-echo sequences. T2* image analysis was performed using custom-written, previously validated software (HIPPO MIOT^®^) [23]. Hepatic T2* values were calculated in a circular region of interest (ROI) of standard dimension, chosen in a homogeneous area of parenchyma [24]. As recommended [25], the liver T2* was converted into liver iron concentration (LIC) by using the Wood’s calibration curve [26]. Three small regions of interest (ROIs) were manually drawn over the pancreatic head, body, and tail, avoiding large blood vessels or ducts and areas involved in susceptibility artefacts from gastric or colic intraluminal gas [27]. Global pancreatic T2* values were calculated as the mean of T2* values from the three regions. The myocardial T2* distribution was mapped into a 16 segment LV model, according to the AHA/ACC model; the global heart T2* value was obtained by averaging all segmental T2* values [28].

### 2.4. Diagnostic Criteria

Based on the presence of HCV antibodies (anti-HCV) and HCV ribonucleic acid (HCV-RNA), a categorization in four groups was performed: negative patients, patients who spontaneously cleared the virus in the first six months of infection, patients who eradicated the virus after the treatment with antiviral therapy obtaining a sustained virological response (SVR), and patients with chronic HCV infection.

Endocrine complications were identified by the following criteria:

- Hypogonadism: no spontaneous puberty or failure to proceed through puberty after the age of 16 years; after puberty, in females, loss of menses before the age 45 years and in males reduced libido, impotence, low levels of gonadotropin, and free and total testosterone;
- Hypothyroidism: high serum thyrotropin concentration and normal or reduced free thyroxine levels (primary form), normal or low serum thyrotropin concentration, and reduced free thyroxine levels (central form);
- Hypoparathyroidism: low serum calcium concentration, increased serum phosphate, low serum parathyroid hormone or, if normal, inappropriate for the calcium level;
- Diabetes mellitus: fasting plasma glucose ≥ 126 mg/dL or 2-h plasma glucose ≥ 200 mg/dL during an oral glucose tolerance test or in patients with classic symptoms of hyperglycemia or hyperglycemic crisis, a random plasma glucose ≥ 200 mg/dL [29].

Bone mineral densities in the lumbar spine and femoral neck were measured by Dual X-ray Absorptiometry (DEXA) and were expressed as T-scores. According to the WHO report, a T-score between −1 and <2.5 indicated osteopenia, and a T-score < −2.5 indicated osteoporosis [30].

Heart failure was diagnosed by clinicians based on symptoms, signs, and instrumental findings according to the current guidelines [31]. Arrhythmias were diagnosed only if ECG documented and requiring specific medication. Arrhythmias were classified according to the AHA/ACC guidelines [32].

Only complications clinically active in the last year were considered.

An LIC ≥ 3 mg/g/dw was considered indicating a significant hepatic iron load [33]. Twenty-six milliseconds (ms) was previously demonstrated to be the lowest threshold of normal T2* pancreatic values [21]. A global heart T2* value < 20 ms was considered as a conservative cut off for significant myocardial iron overload (MIO) [34].

The compliance was collected by the investigators of the thalassemia center, and based on the correspondence between the patient’s actual dosing and the prescribed regimen, it was defined as excellent (>80%), good (60–80%), and insufficient (<60%).

### 2.5. Statistical Analysis

All data were analyzed using the SPSS version 27.0 statistical package. Continuous variables were described as mean ± standard deviation (SD). Categorical variables were expressed as frequencies and percentages.

The normality of distribution of the parameters was assessed by using the Kolmogorov–Smirnov test.

For continuous values with normal distribution, comparisons between groups were made by the independent-samples *t*-test (for two groups) or one-way ANOVA (for more than two groups). First, Levene’s test was applied to verify the homogeneity of variances (homoscedasticity). When the significance level of Levene’s test was <0.05 and homoscedasticity could not be assumed, the Welch statistic was used. Wilcoxon’s signed rank test and the Kruskal–Wallis test were applied for continuous values with non-normal distributions. The Bonferroni adjustment was used in all pairwise comparisons. The χ2 testing was performed for noncontinuous variables.

Spearman’s correlation analysis was performed.

Logistic regression was used to evaluate the odds ratio (OR) with 95% confidence intervals (CI). The OR was used to compare the odds for two groups.

In all tests, a two-tailed probability value of 0.05 was considered statistically significant.

## 3. Results

### 3.1. Patient Characteristics

Out of the 114 TM patients followed in our center, 103 (90.4%) were included in this study. Mean age was 40.03 ± 9.15 years (range: 18–65 years), and 59 (57.3%) patients were females. Mean age at the first blood transfusion was 15.30 ± 15.64 months.

Mean serum ferritin levels in the whole study populations were 811.41 ± 500.21 ng/mL. Serum ferritin levels were not correlated with age (R = −0.145; *p* = 0.144) and were comparable between males and females (737.11 ± 418.21 ng/mL vs. 866.81 ± 550.49 ng/mL; *p* = 0.319.)

Three groups were identified based on the mean serum ferritin levels: <500 ng/mL (group 0; N = 32), between 500 and 1000 ng/mL (group 1; N = 43), and between 1000 and 2500 ng/mL (group 2; N = 28).

### 3.2. Serum Ferritin and Clinical Correlates

The demographic, clinical and hematochemical profiles of the three groups are shown in Table 1. No significant differences in terms of age, sex, starting age for regular transfusions or chelation, and frequency of splenectomy were detected. All the evaluated biochemical and hematological parameters were comparable among the groups, with the exception of the triglycerides, which resulted significantly lower in the group 0 than in the group 2 (*p* = 0.033).

Frequency of all complications was comparable among the three groups.

### 3.3. Serum Ferritin and Iron Overload

T2* MRI was performed in 85 patients, of which 26 (30.6%) showed hepatic IO. Specifically, 16 (18.8%) patients had mild IO (MRI LIC between 3 and 7 mg/g/dw), 7 (8.2%) patients had moderate IO (MRI LIC between 7 and 15 mg/g/dw), and 3 (3.5%) patients had heavy IO (MRI LIC ≥ 15 mg/g/dw). Pancreatic and significant myocardial IO were detected, respectively, in 77 (90.6%) and in 5 (5.9%) patients. All patients with significant myocardial IO had pancreatic IO, while one showed a normal MRI LIC value.

Table 2 shows the comparison of MRI data among the three groups of patients: 25 in group 0, 37 in group 1, and 23 in group 2.

**Figure 1:**
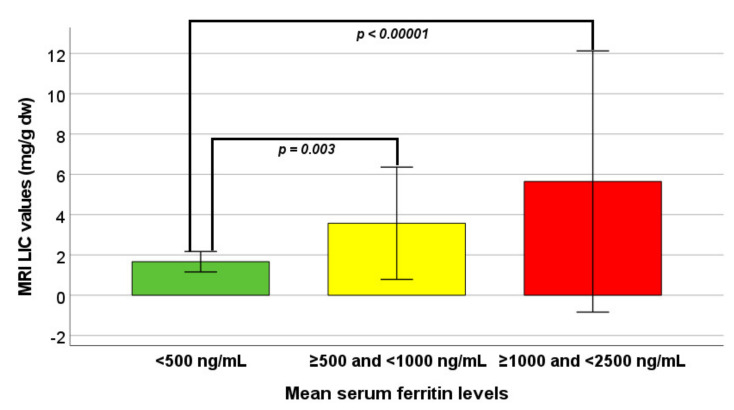
Comparison of MRI LIC values among the three groups identified on the basis of mean serum ferritin levels.

MRI LIC values were significantly different among the three groups. Specifically, patients in group 0 had significantly lower MRI LIC values than patients in group 1 (*p* = 0.003) and in group 2 (*p* < 0.0001) (Figure 1). Hepatic IO was significantly more frequent in group 0 versus both groups 1 and 2 (*p* = 0.006 and *p* = 0.003, respectively). The logistic regression showed a significantly higher risk of hepatic iron overload in group 1 vs. group 0 (OR 14.61, CI 1.78–120.23; *p* = 0.013) as well as in group 2 vs. group 0 (OR 22.00, CI 2.53–190.99; *p* = 0.005).

Globally, MRI LIC values were significantly correlated with mean serum ferritin levels (R = 0.508; *p* < 0.0001), but the correlation was not maintained within each single group [(group 0: R = 0.179; *p* = 0.393), (group 1: R = 0.102; *p* = 0.549), and (group 2: R = 0.295; *p* = 0.171)].

Pancreatic IO was comparable among the three groups.

No patient with a serum ferritin level < 500 ng/mL had significant myocardial IO. The global chi-square test for the comparison among the three groups in terms of frequency of myocardial iron overload yielded a significant difference, but no Bonferroni adjusted pairwise comparison reach statistical significance.

### 3.4. Serum Ferritin and Chelation Therapy

**Figure 2:**
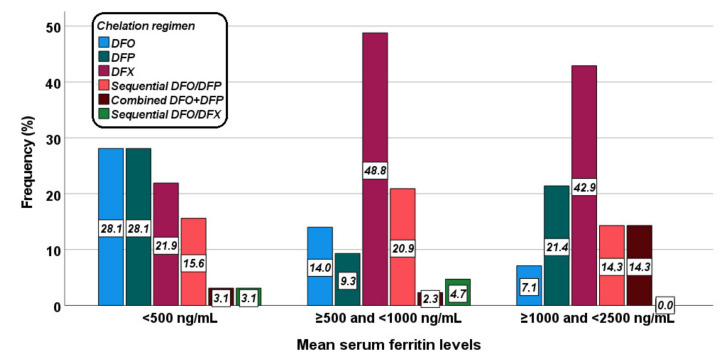
Distribution of the chelation regimens in the three groups identified based on mean serum ferritin levels.

Figure 2 shows the distribution of the different chelation regimens in the three groups. The difference was at the limits of the statistical significance (*p* = 0.053).

Table 3 shows the correlation between serum ferritin levels and IO in different organs for each chelation treatment. In the DFO group a significant correlation was detected between serum ferritin levels and MRI LIC values, and the two (14.3%) patients with hepatic iron overload had a mean serum ferritin value > 500 ng/mL. All patients had pancreatic IO, while no patient had significant myocardial IO. In the DFP group the association between serum ferritin levels and MRI LIC values did not reach statistical significance and out of the eight (53.3%) patients with hepatic IO, one was in group 0 and four were in group 1. The single patient without pancreatic IO was in group 2 while no patient had significant myocardial IO. In the DFX group, a significant correlation was detected between serum ferritin levels and MRI LIC values and all nine (28.1%) patients with hepatic iron overload had a mean serum ferritin value > 500 ng/mL. The three (9.4%) patients with significant myocardial IO had a mean serum ferritin value > 1000 ng/mL. In the group treated with sequential or combined DFO and DFP, serum ferritin level results directly correlated with MRI LIC values and inversely correlated with global heart T2* values. The seven (33.1%) patients with hepatic IO as well as the two (9.5%) patients with significant myocardial IO had a mean serum ferritin value > 500 ng/mL. All patients had pancreatic IO.

The frequency of patients with a good/excellent compliance was 100% in both groups 0 and 1 and 96.4% in group 2 (*p* = 0.256).

### 3.5. Patients with Serum Ferritin Levels ≥ 2500 ng/mL

Mean serum ferritin levels in the 11 excluded patients were 4599.64 ± 3954.22 ng/mL (range: 2513–16,000 ng/mL). Of these patients, 10 (90.4%) had hepatic iron overload, all had pancreatic iron overload, and 4 (36.4%) had myocardial iron overload.

Out of these patients, six were treated with combined or sequential DFO + DFP, three with DFX, three with DFX, one with DFP, and one with sequential DFO/DFX.

The 54.5% of patients with serum ferritin levels ≥ 2500 ng/mL had an insufficient compliance to the chelation therapy. This frequency was significantly higher compared to the patients (1.0%) with serum ferritin levels < 2500 ng/mL (*p* < 0.0001).

## 4. Discussion

The use of intensive and tailored chelation therapy according to the MRI findings has considerably improved the scenario for patients with TDT over the last decades [35,36]. Nevertheless, very few data are available in the literature about the current status of iron overload among TDT patients, particularly regarding the pancreatic site, and the current strategies of chelation in the real life.

The first findings to stress in this study are that the compliance to the chelation therapy plays a vital role in reaching and maintaining serum ferritin levels below 2500 ng/mL and that the proportion of individuals with significant hepatic and myocardial IO was only 30% and 5.9%, respectively. Interestingly, while most of our TDT patients have normal myocardial and hepatic iron, a significant proportion showed simultaneous pancreatic IO. These data are in line with previous observations demonstrating pancreatic IO as a widespread phenomenon among adult patients with TDT [2,11]. However, due to the cross-sectional nature of this study we cannot exclude that pancreatic IO is present long-term in the IO history of the patients and that the pancreas is a kind of “sanctuary compartment” for common iron chelating agents. Prospective assessments are required to establish the benefit in removing pancreatic IO by further intensifying chelation therapy as observed in the seminal study of Farmakis et al., following the use of the combined therapy with deferiprone and desferrioxamine [37]. Few other data are available in the literature about the iron chelation treatment and the aim of normalizing iron depot. Kolnagou et al., using the same protocol to reach normal iron depots, showed that normal ferritin levels could be maintained using deferiprone monotherapy without serious toxicity and symptoms of over-chelation [38]. More recently, a report from Pinna et al. described the clinical management of patients with thalassemia major who reached serum ferritin values < 500 ng/mL in the last 15 years [39]. In this retrospective evaluation, patients under DFX or DFX plus DFO combination were also described, but a general tolerability and side effects that were not significant in patients who stayed under 500 ng/mL were reported, apart from the description of rachiodynia whose appearance was not judged as definitely related to iron depletion by the authors.

Our data stratified by groups of ferritin levels showed a significant association between low ferritin levels and the absence of hepatic and cardiac iron but not of pancreatic iron. We observed a linear association between LIC and ferritin levels. No patient with a serum ferritin level < 500 ng/mL had significant myocardial IO, but pancreatic IO was present in all but one patient. We did not observe signs of hematological/renal toxicity in any patients. Looking at morbidity, the prevalence of all complications was comparable among the three groups, reflecting a paradoxical substantial equivalency in the burden of the disease, which likely reflected the different history of the disease and could be not emphasized by the cross-sectional study design. Furthermore, the increased triglyceride levels among patients with the highest level of ferritin may reflect the influence on ferritin levels by disorders of lipidic metabolism and/or the presence of hepatic steatosis [40,41]. Consequently, the higher the triglycerides levels, the higher is the chance to encounter high ferritin levels, reflecting not only iron overload but also hepatic steatosis. Hence, natural directions for future research may include the simultaneous evaluation of the fat component during an MRI liver evaluation.

However, our analysis also revealed a noteworthy proportion of patients (65.0%) with ferritin < 500 ng/mL and/or normal LIC; this condition was maintained not only by all single chelators but also by DFP and DFO in sequential or combination therapy without any side effects. These data are in accordance with previous prospective observations from multicentric studies where all these chelation regimens were capable of stabilizing normal iron burdens [9,10]. Therefore, taking in account that these data reflect at least 12 months of observations on continuous chelation, we could propose that to target patients of normal or near-normal serum ferritin levels may be feasible and safe under several chelation regimens. However, this cross-sectional study lacks an intention to treat analysis and does not reveal the quota of patients in which this achievement was limited or had failed due to side effects, as it may happen for transient proteinuria/renal dysfunction in patients treated with DFX [42].

Regardless of the chelation regimen and the relationships between serum ferritin and LIC, this study reinforces the previous observation that in the patients treated with DFP the association between serum ferritin levels and MRI LIC values is not so rigorous and did not reach statistical significance [43]. Moreover, patients on DFP monotherapy tend to have higher LICs at comparable serum ferritin levels, with respect to patients under DFO or DFX therapies. On the other hand, in patients treated with DFO or DFX, normal ferritin levels predicted no hepatic or no cardiac iron. As a consequence, it is plausible that patients under these chelators, in case of persistence of normal ferritin level, could undergo MRI regular monitoring for LIC and myocardial IO at delayed frequencies with respect to suggestions by the current guidelines [44,45]. Further prospective studies are needed to validate this hypothesis.

Serum ferritin < 500 ng/mL appears to be achievable and safe for several TDT patients, as it is not associated with an alteration in the main hematological parameters. Ferritin levels can be considered a reliable surrogate marker for body iron load in TDT patients, although their elevation may reflect phenomena not linked to iron metabolism, such as inflammation, liver damage, and steatohepatitis. Overall, our data suggest that a strategy to reach ferritin < 500 ng/mL could be achievable and safe for several adult TDT patients. However, this management should be directed only to adult patients, while special situations such as childhood and transitional age still merit separate and specific evaluations.

This target is also associated with the absence of significant cardiac iron and significantly lower LIC and triglycerides that are well-demonstrated markers for cardiac and liver complications. Thus, a serum ferritin trend near to the normality may be beneficial for TDT patients, even though it requires careful management and monitoring to avoid over-chelation, and it should be performed only in centers specialized in the care of the hemoglobinopathies. Otherwise, the recommendation to interrupt chelation therapy when the ferritin level is below 1000 ng/mL should be considered still valid [46]. Further, multicenter studies are needed to confirm these findings in larger series and to assess the impact of this strategy on overall morbidity in younger patients.

## Figures and Tables

**Table 1:** Comparison of demographic, clinical, hematological, and biochemical characteristics among the three groups.

	Mean Serum Ferritin < 500 ng/mL (N = 32)	Mean Serum Ferritin ≥ 500 and <1000 ng/mL (N = 43)	Mean Serum Ferritin ≥ 1000 and <2500 ng/mL (N = 28)	*p*
Demographic and clinical characteristics				
Age (years)	41.87 ± 7.78	39.88 ± 8.93	38.15 ± 10.74	0.292
Females, N (%)	17 (53.1)	26 (60.5)	16 (57.1)	0.817
Regular transfusions starting age (months)	16.47 ± 18.08	13.28 ± 13.96	19.79 ± 28.96	0.192
Chelation starting age (years)	7.33 ± 5.12	5.82 ± 4.38	6.27 ± 6.76	0.255
Genotype, N (%)				0.998
β+β+	5 (15.6)	7 (16.3)	5 (17.9)	
β0β+	15 (46.9)	20 (46.5)	12 (42.9)	
β0β0	12 (37.5)	16 (37.2)	11 (39.3)	
Splenectomy, N (%)	21 (65.6)	22 (51.2)	13 (46.4)	0.283
HCV infection, N (%)				0.515
Negative	10 (31.3)	11 (25.6)	10 (35.7)	
Spontaneously eradicated	11 (34.4)	15 (34.9)	8 (28.6)	
Eradicated after treatment	5 (15.6)	14 (32.6)	6 (24.0)	
Chronic	6 (18.8)	3 (7.0)	4 (14.3)	
Hematological parameters				
Mean hemoglobin (g/dL)	9.93 ± 0.34	9.78 ± 0.38	9.78 ± 0.41	0.265
Alanine aminotransferase (U/L)	31.59 ± 28.07	21.63 ± 11.07	29.04 ± 20.31	0.274
Aspartate aminotransferase (U/L)	29.25 ± 24.15	22.23 ± 8.67	25.32 ± 10.12	0.274
Gamma-glutamyl transpeptidase (U/L)	29.59 ± 23.29	21.35 ± 18.11	29.11 ± 17.47	0.053
Creatinine (mg/dL	0.67 ± 0.16	0.67 ± 0.14	1.02 ± 1.26	0.243
Uric acid (mg/dL)	4.47 ± 0.96	4.02 ± 1.03	4.34 ± 0.81	0.155
Total cholesterol (mg/dL)	106.71 ± 27.86	111.51 ± 25.95	115.39 ± 28.75	0.487
Triglycerides (mg/dL)	91.43 ± 42.47	99.37 ± 44.30	140.59 ± 92.43	0.025
High-density lipoprotein cholesterol (mg/dL)	38.15 ± 14.65	41.34 ± 13.26	36.87 ± 13.52	0.408
Complications				
Hepatic cirrhosis, N (%)	0 (0.0)	2 (4.7)	0 (0.0)	0.241
Hypogonadism, N (%)	17 (53.1)	23 (53.5)	12 (42.9)	0.639
Hypothyroidism, N (%)	7 (21.9)	11 (25.6)	7 (25.0)	0.929
Hypoparathyroidism, N (%)	1 (3.1)	1 (2.3)	1 (3.6)	0.951
Diabetes mellitus, N (%)	5 (15.6)	3 (7.0)	3 (10.7)	0.487
Osteopenia/osteoporosis, N (%)	24 (75.0)	33 (76.7)	21 (75.0)	0.979
Hearing loss, N (%)	7 (21.9)	11/32 (26.1)	6/26 (23.2)	0.904
Ophthalmic diseases, N (%)	0 (0.0)	0/41 (0.0)	0/25 (0.0)	-
Heart failure, N (%)	1 (3.1)	0 (0.0)	0 (0.0)	0.326
Arrhythmias, N (%)	2 (6.3)	1 (2.3)	1 (3.6)	0.681

N = number, HCV = hepatitis c virus.

**Table 2:** Hepatic, pancreatic, and cardiac iron overload in patients in whom an MRI scan was performed.

	Mean Serum Ferritin < 500 ng/mL (N = 25)	Mean Serum Ferritin ≥ 500 and <1000 ng/mL (N = 37)	Mean Serum Ferritin ≥ 1000 and <2500 ng/mL (N = 23)	*p*
MRI LIC (mg/g dw)	1.67 ± 0.51	3.57 ± 2.79	5.65 ± 6.48	<0.0001
MRI LIC ≥ 3 mg/g dw, N (%)	1 (4.0)	14 (37.8)	11 (47.8)	0.002
Global pancreas T2* (ms)	9.34 ± 8.24	11.29 ± 8.29	9.92 ± 8.72	0.479
Global pancreas T2* < 26 ms, N (%)	23 (92.0)	34 (91.9)	20 (87.0)	0.783
Global heart T2* (ms)	42.51 ± 4.75	38.79 ± 8.16	34.91 ± 11.64	0.048
Global heart T2* < 20 ms, N (%)	0 (0.0)	1 (2.7)	4 (17.4)	0.021

N = number, MRI = magnetic resonance imaging, LIC = liver iron concentration.

**Table 3:** Correlation between serum ferritin levels and hepatic, pancreatic, and cardiac iron overload in each group identified based on the chelation treatment.

	Mean Serum Ferritin Levels	MRI LIC	Global Pancreas T2*	Global Heart T2*
DFO therapy (N = 14)				
Mean value	608.14 ± 326.68 ng/mL	1.88 ± 1.03 mg/g dw	8.63 ± 5.39 ms	40.71 ± 7.43 ms
Correlation with ferritin		R = 0.697 *p* = 0.006	R = −0.341 *p* = 0.233	R = −0.393 *p* = 0.164
DFP therapy (N = 15)				
Mean value	838.67 ± 612.19 ng/mL	5.08 ± 4.17 mg/g dw	8.37 ± 6.49 ms	40.48 ± 4.79 ms
Correlation with ferritin		R = 0.463 *p* = 0.082	R = 0.297 *p* = 0.282	R = 0.145 *p* = 0.606
DFX therapy (N = 32)				
Mean value	875.06 ± 468.48 ng/mL	4.12 ± 5.52 mg/g dw	13.74 ± 10.64 ms	38.44 ± 9.54 ms
Correlation with ferritin		R = 0.491 *p* = 0.004	R = −0.092 *p* = 0.623	R = −0.243 *p* = 0.181
Sequential or combined DFO/DFP (N = 21)				
Mean value	863.57 ± 510.39 ng/mL	3.10 ± 2.20 mg/g dw	7.47 ± 4.43 ms	37.09 ± 11.09 ms
Correlation with ferritin		R = 0.443 *p* = 0.044	R = 0.045 *p* = 0.847	R = −0.622 *p* = 0.003

MRI = magnetic resonance imaging, LIC = liver iron concentration, N = number, DFO = desferrioxamine, DFP = deferiprone, DFX = deferasirox.

## Data Availability

Data will be made available upon request to the corresponding author.

## References

[1] Pepe A., Meloni A., Rossi G., Midiri M., Missere M., Valeri G., Sorrentino F., D’Ascola D.G., Spasiano A., Filosa A. (2018). Prediction of cardiac complications for thalassemia major in the widespread cardiac magnetic resonance era: A prospective multicentre study by a multi-parametric approach. Eur. Heart J. Cardiovasc. Imaging.

[2] Pepe A., Pistoia L., Gamberini M.R., Cuccia L., Peluso A., Messina G., Spasiano A., Allo M., Bisconte M.G., Putti M.C. (2020). The Close Link of Pancreatic Iron With Glucose Metabolism and With Cardiac Complications in Thalassemia Major: A Large, Multicenter Observational Study. Diabetes Care.

[3] Borgna-Pignatti C., Rugolotto S., De Stefano P., Zhao H., Cappellini M.D., Del Vecchio G.C., Romeo M.A., Forni G.L., Gamberini M.R., Ghilardi R. (2004). Survival and complications in patients with thalassemia major treated with transfusion and deferoxamine. Haematologica.

[4] Modell B., Khan M., Darlison M., Westwood M.A., Ingram D., Pennell D.J. (2008). Improved survival of thalassaemia major in the UK and relation to T2* cardiovascular magnetic resonance. J. Cardiovasc. Magn. Reson..

[5] Tanner M.A., Galanello R., Dessi C., Smith G.C., Westwood M.A., Agus A., Roughton M., Assomull R., Nair S.V., Walker J.M. (2007). A randomized, placebo-controlled, double-blind trial of the effect of combined therapy with deferoxamine and deferiprone on myocardial iron in thalassemia major using cardiovascular magnetic resonance. Circulation.

[6] Pennell D.J., Berdoukas V., Karagiorga M., Ladis V., Piga A., Aessopos A., Gotsis E.D., Tanner M.A., Smith G.C., Westwood M.A. (2006). Randomized controlled trial of deferiprone or deferoxamine in beta-thalassemia major patients with asymptomatic myocardial siderosis. Blood.

[7] Berdoukas V., Chouliaras G., Moraitis P., Zannikos K., Berdoussi E., Ladis V. (2009). The efficacy of iron chelator regimes in reducing cardiac and hepatic iron in patients with thalassaemia major: A clinical observational study. J. Cardiovasc. Magn. Reson..

[8] Pennell D.J., Porter J.B., Piga A., Lai Y., El-Beshlawy A., Belhoul K.M., Elalfy M., Yesilipek A., Kilinc Y., Lawniczek T. (2014). A 1-year randomized controlled trial of deferasirox vs. deferoxamine for myocardial iron removal in beta-thalassemia major (CORDELIA). Blood.

[9] Pepe A., Meloni A., Rossi G., Cuccia L., D’Ascola G.D., Santodirocco M., Cianciulli P., Caruso V., Romeo M.A., Filosa A. (2013). Cardiac and hepatic iron and ejection fraction in thalassemia major: Multicentre prospective comparison of combined deferiprone and deferoxamine therapy against deferiprone or deferoxamine monotherapy. J. Cardiovasc. Magn. Reson..

[10] Pepe A., Meloni A., Pistoia L., Cuccia L., Gamberini M.R., Lisi R., D’Ascola D.G., Rosso R., Allo M., Spasiano A. (2018). MRI multicentre prospective survey in thalassaemia major patients treated with deferasirox versus deferiprone and desferrioxamine. Br. J. Haematol..

[11] Noetzli L.J., Mittelman S.D., Watanabe R.M., Coates T.D., Wood J.C. (2012). Pancreatic iron and glucose dysregulation in thalassemia major. Am. J. Hematol..

[12] Meloni A., Restaino G., Missere M., De Marchi D., Positano V., Valeri G., Giuseppe D’Ascola D., Peluso A., Caterina Putti M., Lendini M. (2015). Pancreatic iron overload by T2* MRI in a large cohort of well treated thalassemia major patients: Can it tell us heart iron distribution and function?. Am. J. Hematol..

[13] Davis B.A., O’Sullivan C., Jarritt P.H., Porter J.B. (2004). Value of sequential monitoring of left ventricular ejection fraction in the management of thalassemia major. Blood.

[14] Gabutti V., Piga A. (1996). Results of long-term iron-chelating therapy. Acta Haematol..

[15] Olivieri N.F., Nathan D.G., MacMillan J.H., Wayne A.S., Liu P.P., McGee A., Martin M., Koren G., Cohen A.R. (1994). Survival in medically treated patients with homozygous beta-thalassemia. N. Engl. J. Med..

[16] Musallam K.M., Angastiniotis M., Eleftheriou A., Porter J.B. (2013). Cross-talk between available guidelines for the management of patients with beta-thalassemia major. Acta Haematol..

[17] Pepe A., Positano V., Santarelli F., Sorrentino F., Cracolici E., De Marchi D., Maggio A., Midiri M., Landini L., Lombardi M. (2006). Multislice multiecho T2* cardiovascular magnetic resonance for detection of the heterogeneous distribution of myocardial iron overload. J. Magn. Reson. Imaging.

[18] Ramazzotti A., Pepe A., Positano V., Rossi G., De Marchi D., Brizi M.G., Luciani A., Midiri M., Sallustio G., Valeri G. (2009). Multicenter validation of the magnetic resonance t2* technique for segmental and global quantification of myocardial iron. J. Magn. Reson. Imaging.

[19] Meloni A., De Marchi D., Pistoia L., Grassedonio E., Peritore G., Preziosi P., Restaino G., Righi R., Riva A., Renne S. (2019). Multicenter validation of the magnetic resonance T2* technique for quantification of pancreatic iron. Eur. Radiol..

[20] Positano V., Salani B., Pepe A., Santarelli M.F., De Marchi D., Ramazzotti A., Favilli B., Cracolici E., Midiri M., Cianciulli P. (2009). Improved T2* assessment in liver iron overload by magnetic resonance imaging. Magn. Reson. Imaging.

[21] Restaino G., Meloni A., Positano V., Missere M., Rossi G., Calandriello L., Keilberg P., Mattioni O., Maggio A., Lombardi M. (2011). Regional and global pancreatic T*(2) MRI for iron overload assessment in a large cohort of healthy subjects: Normal values and correlation with age and gender. Magn. Reson. Med..

[22] Meloni A., Positano V., Pepe A., Rossi G., Dell’Amico M., Salvatori C., Keilberg P., Filosa A., Sallustio G., Midiri M. (2010). Preferential patterns of myocardial iron overload by multislice multiecho T*2 CMR in thalassemia major patients. Magn. Reson. Med..

[23] Positano V., Pepe A., Santarelli M.F., Scattini B., De Marchi D., Ramazzotti A., Forni G., Borgna-Pignatti C., Lai M.E., Midiri M. (2007). Standardized T2* map of normal human heart in vivo to correct T2* segmental artefacts. NMR Biomed..

[24] Meloni A., Luciani A., Positano V., De Marchi D., Valeri G., Restaino G., Cracolici E., Caruso V., Dell’amico M.C., Favilli B. (2011). Single region of interest versus multislice T2* MRI approach for the quantification of hepatic iron overload. J. Magn. Reson. Imaging.

[25] Meloni A., Rienhoff H.Y., Jones A., Pepe A., Lombardi M., Wood J.C. (2013). The use of appropriate calibration curves corrects for systematic differences in liver R2* values measured using different software packages. Br. J. Haematol..

[26] Wood J.C., Enriquez C., Ghugre N., Tyzka J.M., Carson S., Nelson M.D., Coates T.D. (2005). MRI R2 and R2* mapping accurately estimates hepatic iron concentration in transfusion-dependent thalassemia and sickle cell disease patients. Blood.

[27] Meloni A., De Marchi D., Positano V., Neri M.G., Mangione M., Keilberg P., Lendini M., Cirotto C., Pepe A. (2015). Accurate estimate of pancreatic T2* values: How to deal with fat infiltration. Abdom. Imaging.

[28] Meloni A., Positano V., Ruffo G.B., Spasiano A., D’Ascola D.G., Peluso A., Keilberg P., Restaino G., Valeri G., Renne S. (2015). Improvement of heart iron with preserved patterns of iron store by CMR-guided chelation therapy. Eur. Heart J. Cardiovasc. Imaging.

[29] (2011). American Diabetes Association. Standards of Medical Care in Diabetes—2011. Diabetes Care.

[30] WHO (2003). Prevention and Management of Osteoporosis.

[31] McDonagh T.A., Metra M., Adamo M., Gardner R.S., Baumbach A., Bohm M., Burri H., Butler J., Celutkiene J., Chioncel O. (2021). 2021 ESC Guidelines for the diagnosis and treatment of acute and chronic heart failure. Eur. Heart J..

[32] Buxton A.E., Calkins H., Callans D.J., DiMarco J.P., Fisher J.D., Greene H.L., Haines D.E., Hayes D.L., Heidenreich P.A., Miller J.M. (2006). ACC/AHA/HRS 2006 key data elements and definitions for electrophysiological studies and procedures: A report of the American College of Cardiology/American Heart Association Task Force on Clinical Data Standards (ACC/AHA/HRS Writing Committee to Develop Data Standards on Electrophysiology). Circulation.

[33] Angelucci E., Brittenham G.M., McLaren C.E., Ripalti M., Baronciani D., Giardini C., Galimberti M., Polchi P., Lucarelli G. (2000). Hepatic iron concentration and total body iron stores in thalassemia major. N. Engl. J. Med..

[34] Positano V., Meloni A., Santarelli M.F., Gerardi C., Bitti P.P., Cirotto C., De Marchi D., Salvatori C., Landini L., Pepe A. (2015). Fast generation of T2* maps in the entire range of clinical interest: Application to thalassemia major patients. Comput. Biol. Med..

[35] Ricchi P., Ammirabile M., Spasiano A., Costantini S., Cinque P., Di Matola T., Pagano L., Prossomariti L. (2010). Combined chelation therapy in thalassemia major with deferiprone and desferrioxamine: A retrospective study. Eur. J. Haematol..

[36] Pepe A., Meloni A., Rossi G., Dell’Amico M.C., Spasiano A., Capra M., Cianciulli P., Caruso V., Favilli B., Cracolici E. (2011). A T2* MRI prospective survey on heart iron in thalassemia major patients treated with deferasirox versus deferiprone and desferrioxamine in monotherapy. J. Cardiovasc. Magn. Reson..

[37] Farmaki K., Tzoumari I., Pappa C., Chouliaras G., Berdoukas V. (2010). Normalisation of total body iron load with very intensive combined chelation reverses cardiac and endocrine complications of thalassaemia major. Br. J. Haematol..

[38] Kolnagou A., Kleanthous M., Kontoghiorghes G.J. (2011). Efficacy, compliance and toxicity factors are affecting the rate of normalization of body iron stores in thalassemia patients using the deferiprone and deferoxamine combination therapy. Hemoglobin.

[39] Pinna F., Carta R., Morittu M., Dessi C., Moi P., Leoni G., Foschini M.L., Defraia E., Zappu A., Origa R. (2015). Thalassemia Major: Who Is Afraid of Serum Ferritin below 500 mug/l?. Acta Haematol..

[40] Jehn M., Clark J.M., Guallar E. (2004). Serum ferritin and risk of the metabolic syndrome in U.S. adults. Diabetes Care.

[41] Ricchi P., Meloni A., Spasiano A., Costantini S., Pepe A., Cinque P., Filosa A. (2018). The impact of liver steatosis on the ability of serum ferritin levels to be predictive of liver iron concentration in non-transfusion-dependent thalassaemia patients. Br. J. Haematol..

[42] Ponticelli C., Musallam K.M., Cianciulli P., Cappellini M.D. (2010). Renal complications in transfusion-dependent beta thalassaemia. Blood Rev..

[43] Ang A.L., Shah F.T., Davis B.A., Thomas A., Murugachandran G., Kumuradevan J., Garbowksi M.W., Porter J.B. (2010). Deferiprone is Associated with Lower Serum Ferritin (SF) Relative to Liver Iron Concentration (LIC) Than Deferoxamine and Deferasirox- Implications for Clinical Practice. Blood.

[44] Pennell D.J., Udelson J.E., Arai A.E., Bozkurt B., Cohen A.R., Galanello R., Hoffman T.M., Kiernan M.S., Lerakis S., Piga A. (2013). Cardiovascular function and treatment in beta-thalassemia major: A consensus statement from the American Heart Association. Circulation.

[45] Shah F.T., Porter J.B., Sadasivam N., Kaya B., Moon J.C., Velangi M., Ako E., Pancham S. (2021). Guidelines for the monitoring and management of iron overload in patients with haemoglobinopathies and rare anaemias. Br. J. Haematol..

[46] Taher A.T., Musallam K.M., Cappellini M.D. (2021). beta-Thalassemias. N. Engl. J. Med..

